# Activation of AMP-activated protein kinase attenuates hepatocellular carcinoma cell adhesion stimulated by adipokine resistin

**DOI:** 10.1186/1471-2407-14-112

**Published:** 2014-02-20

**Authors:** Chen-Chieh Yang, Shun-Fu Chang, Jian-Kang Chao, Yi-Liang Lai, Wei-En Chang, Wen-Hsiu Hsu, Wu-Hsien Kuo

**Affiliations:** 1 Division of Gastroenterology, Department of Internal Medicine, Armed-Forces Hualien General Hospital, Hualien 97144, Taiwan; 2 Mennonite Christian Hospital, Hualien 97059, Taiwan; 3 Biophotonics & Molecular Imaging Research Center, National Yang Ming University, Taipei 11221, Taiwan; 4 Department of Psychiatry, Yuli branch, Taipei Veterans General Hospital, Hualien 98142, Taiwan; 5 Department of Health Administration, Tuz Chi College of Technology, Hualien, Taiwan; 6 Division of Gastroenterology, Department of Internal Medicine, Armed-Forces Taichung General Hospital, Taiping District, Taichung City 41168, Taiwan; 7 School of Medicine, National Defense Medical Center, Taipei 11490, Taiwan

**Keywords:** AMP-activated protein kinase, Hepatocellular carcinoma, Intercellular adhesion molecule-1, Resistin, Vascular cell adhesion molecule-1

## Abstract

**Background:**

Resistin, adipocyte-secreting adipokine, may play critical role in modulating cancer pathogenesis. The aim of this study was to investigate the effects of resistin on HCC adhesion to the endothelium, and the mechanism underlying these resistin effects.

**Methods:**

Human SK-Hep1 cells were used to study the effect of resistin on intercellular adhesion molecule-1 (ICAM-1) and vascular cell adhesion molecule-1 (VCAM-1) expressions as well as NF-κB activation, and hence cell adhesion to human umbilical vein endothelial cells (HUVECs). 5-Aminoimidazole-4-carboxamide 1-β-D-ribofuranoside (AICAR), an AMP-activated protein kinase (AMPK) activator, was used to determine the regulatory role of AMPK on HCC adhesion to the endothelium in regard to the resistin effects.

**Results:**

Treatment with resistin increased the adhesion of SK-Hep1 cells to HUVECs and concomitantly induced NF-κB activation, as well as ICAM-1 and VCAM-1 expressions in SK-Hep1 cells. Using specific blocking antibodies and siRNAs, we found that resistin-induced SK-Hep1 cell adhesion to HUVECs was through NF-κB-regulated ICAM-1 and VCAM-1 expressions. Moreover, treatment with AICAR demonstrated that AMPK activation in SK-Hep1 cells significantly attenuates the resistin effect on SK-Hep1 cell adhesion to HUVECs.

**Conclusions:**

These results clarify the role of resistin in inducing HCC adhesion to the endothelium and demonstrate the inhibitory effect of AMPK activation under the resistin stimulation. Our findings provide a notion that resistin play an important role to promote HCC metastasis and implicate AMPK may be a therapeutic target to against HCC metastasis.

## Background

Hepatocellular carcinoma (HCC), one of the most frequent visceral neoplasms worldwide, is a common fatal malignant tumor characterized by a high incidence of recurrence, development of resistance to chemotherapy, and low sensitivity to radiation therapy [1,2]. HCC is a primary malignant cancer of the liver. In spite of many well-defined risk factors for HCC, including: hepatitis B and C viral infections, alcoholism and cirrhosis, it has still been shown that 5 ~ 30% of patients with HCC lack a readily identifiable risk factor [3,4]. Recently, epidemiological studies have indicated that obesity, especially abdominal obesity, is a new risk factor of HCC [1,5-8]. Adipose tissue is now broadly recognized as an endocrine organ secreting numerous proteins, also called adipokines, with both physiological and pathophysiological functions [3,9]. Resistin is a novel adipocyte-secreting adipokine, and its activity is implicated in inflammatory processes including: atherosclerosis, rheumatic diseases, nonalcoholic fatty liver disease, and malignancies [10]. Mounting evidence indicates that serum resistin is proportionally related to cancer development, including: breast, gastric, and colorectal cancers as well as lymphoma. It has also been suggested that the expression of resistin in cancer cells is associated with more malignant clinicopathological processes [1,3,10,11]. However, there is still a lack of information about the precise mechanisms of resistin on HCC development, including adhesion and invasion.

Cancer cell metastasis is a major clinical problem and, in the case of most cancers, results in a poor prognosis due to cancer cell development. The maintenance and promotion of cell adhesion are particularly critical processes in cancer cell metastasis. These processes are modulated by the interaction of cells with one another and with their microenvironment. Moreover, cell adhesion molecules (CAMs) facilitate these interactions, and are necessary for all of the metastasis process [12,13]. The intercellular adhesion molecule-1 (ICAM-1) and vascular cell adhesion molecule-1 (VCAM-1) belong to the CAM-immunoglobulin (Ig) gene superfamily of adhesion molecules. They can induce the cancer cell adhesion to the endothelium and are also involved in the immune responses of tumors [14]. The interaction between ICAM-1/VCAM-1 and their respective ligand may facilitate the adhesion of cancer cells to the vascular endothelium, and subsequently aid in the promotion of metastasis. Resistin has been indicated to induce ICAM-1 and VCAM-1 expressions through transcription factor NF-κB in endothelial cells and to initiate the cancer cells and monocyte adhesion [15,16]. However, there is no detailed information about whether resistin induces ICAM-1 and VCAM-1 expressions in cancer cells, and hence promotes its adhesion and invasion of the endothelium.

An energy imbalance underlies many human metabolic diseases, including obesity and cancer. The AMP-activated protein kinase (AMPK) is a highly conserved sensor of cellular energy status and a well-known regulator of cell metabolism [17,18]. It is a heterotrimeric enzyme consisting of an α catalytic subunit and non-catalytic regulatory β and γ subunits. AMPK is activated by a decrease in the ratio of ATP to AMP, which inhibits ATP-consuming metabolic pathways and activates the energy-producing pathways [19]. Recently, AMPK activation has been demonstrated to play an important role in regulating cancer development [17-22]. However, the detailed molecular mechanisms between AMPK activation and cancer metastasis are still controversial, and whether AMPK activation is a critical controller for HCC adhesion and subsequent metastasis is unclear.

In the present study, we investigated the regulatory effects of resistin on HCC adhesion to the endothelium in human SK-Hep1 cells and the mechanism underlying these resistin effects. We found resistin-induced SK-Hep1 cell adhesion to endothelial cells through transcription factor NF-κB activation as well as ICAM-1 and VCAM-1 expressions. Moreover, these effects were attenuated by AMPK activation in SK-Hep1 cells. Our findings provide new insights into the understanding of resistin-regulated the adhesion of HCC to the endothelium and the inhibitory effects of AMPK activation.

## Methods

### Materials

All culture materials were purchased from Gibco (Grand Island, NY, USA). Recombinant human resistin was purchased from R & D Systems (Minneapolis, MN). Polyclonal anti-ICAM-1 and anti-VCAM-1 neutralizing antibodies were obtained from R & D Systems (Minneapolis, MN). ICAM-1-, VCAM-1-specific siRNA and control siRNA (a scrambled negative control containing random DNA sequences) were purchased from Invitrogen (Carlsbad, CA). The sequences of ICAM-1- and VCAM-1-specific siRNA are 5′-CGGCU GGAGC UGUUU GAGAT T-3′ and 5′-GGAGU GAUUU UUCUA UCGGT T-3′, and 5′-AAUGC AACUC UCACC UUAAT T-3′ and 5′-UUAAG GUGAG AGUUG CAUUT T-3′, respectively. Pyrrolidine dithiocarbamate (PDTC, NF-κB inhibitor), SN50, AICAR and all other chemicals of a reagent grade were obtained from Sigma (St. Louis, MO).

### Cell culture

The HCC SK-Hep1 cells were purchased from the Bioresources Collection and Research Center (BCRC) of the Food Industry Research and Development Institute (Hsinchu, Taiwan). Cells were maintained in Dulbecco’s Modified Eagle Medium (DMEM) supplemented with 10% fetal bovine serum (FBS) and 1% penicillin/streptomycin in a CO_2_ incubator at 37°C.

Human umbilical vein endothelial cells (HUVECs) were purchased from Cambrex Bio Science (Walkersville, MD). Cells were cultured in endothelial cell growth medium-2 (EGM-2) and maintained at 37°C in a humidified atmosphere containing 5% CO_2_. HUVECs of passage 2 were used for the experiments.

### Cell adhesion assay

Before the adhesion experiments, cells were cultured in low-serum medium (0.5% FBS) for 12 h and then treated with resistin (50 ng/mL) for 4 h and labeled with 1, 10-dioctadecyl-3, 3, 30, 30-tetramethylindocarbocyanine (DiI; Molecular Probes, Eugene, OR) for 20 min. The labeled cells (2×10^5^ cells/mL) were added to HUVECs and incubated for 1 h. In parallel experiments, SK-Hep1 cells were treated with specific inhibitors, neutralizing antibodies, or transfected with specific siRNAs during resistin stimulation. Non-adherent cells were removed by washing with PBS. The adherent cancer cells on the EC surface were identified and counted in 10 randomly selected microscopic fields (1.37 mm×1.07 mm) under a Nikon Ti-E inverted epifluorescence microscope with ×10 objective, and the adhesion was expressed as a multiple compared to the controls.

### Real-time quantitative PCR

Real-time PCR of three transcripts was performed using an ABI Prism 7900HT with the FastStart DNA SYBR Green I kit (Roche). The designed primers in this study were: ICAM-1 forward primer, 5′-GTGAC ATGCA GCACC TCCTG-3′; ICAM-1 reverse primer, 5′-TCCAT GGTGA TCTCT CCTCA-3′; VCAM-1 forward primer, 5′-CCGGA TTGCT GCTCA GATTG GA-3′; VCAM-1 reverse primer, 5′-AGCGT GGAAT TGGTC CCCTC A-3′; 18S rRNA forward primer, 5′-CGGCG ACGAC CCATT CGAAC-3′; 18S rRNA reverse primer, 5′-GAATC GAACC CTGAT TCCCC GTC-3′. Quantification was performed using the 2^−ΔΔCt^ method (30). All samples were measured in duplicate. The average value of both duplicates was used as the quantitative value.

### ELISA for cell surface ICAM-1 and VCAM-1 expression

ICAM-1 and VCAM-1 expression on the cancer cell surface was measured by cell surface ELISA as previously described [23]. Briefly, SK-Hep1 cells cultured in 96-well plates were fixed by 4% paraformaldehyde. Cell surface ICAM-1 and VCAM-1 expression were assessed using the mouse anti-human ICAM-1 or VCAM-1 mAb, followed by a horseradish-peroxidase-conjugated secondary antibody. Cells were then washed with PBS before the addition of 3,3′,5,5′-tetramethylbenzidine. The reaction was allowed to develop at room temperature before *A*_600_ determination using a plate reader.

### siRNA transfection

For siRNA transfection, SK-Hep1 cells were transfected with the specific ICAM-1 and VCAM-1-siRNA or control siRNA by using an RNAiMAX transfection kit (Invitrogen).

### Transcription factor assays (TF ELISA assays)

Nuclear extracts of cells were prepared by nuclear protein extract kits (Panomics, Redwood City, CA). Equal amounts of nuclear proteins were used for quantitative measurements of NF-κB p65 activation using commercially available ELISA kits (Panomics).

### Statistical analysis

The results are expressed as the mean ± standard error of the mean (SEM). Statistical analysis was determined using an independent Student t-test for two groups of data and analysis of variance (ANOVA) followed by Scheffe’s test for multiple comparisons. *P* values less than 0.05 were considered significant.

## Results

### Resistin induced the adhesions of SK-Hep1 cells to HUVECs

**Figure 1:**
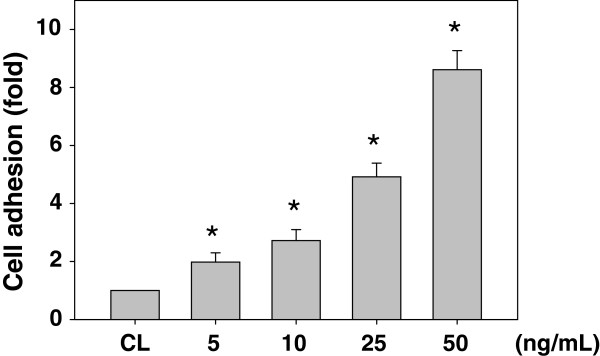
**Resistin induced the adhesions of SK-Hep1 cells to HUVECs.** SK-Hep1 cells were kept as CL or treated with resistin at 5, 10, 25 and 50 ng/mL for 4 h. They were then labeled with DiI and added to confluent monolayers of HUVECs for 1 h. The bar graphs represent the mean ± standard error of the mean (SEM) from four independent experiments. *, *P* < 0.05 *vs*. control cells.

In order to determine the HCC cancer cell adhesion to the endothelium, SK-Hep1 cells were kept as the control or treated with different concentrations of resistin (i.e. 5, 10, 25 and 50 ng/ml) for 4 h and then subsequently marked with the fluorescent cell tracker DiI to test the adhesions of cells to HUVECs. The marked cells were seeded onto the HUVEC monolayers and co-cultured for 1 h. After removal of the non-adherent cells, the remaining adherent cells were examined. Treatment with resistin for 4 h resulted in increased adhesions of SK-Hep1 cells to HUVECs in a dose-dependent manner over the range tested, and the induction reached a level approximately 8 times the untreated control under 50 ng/mL of resistin treatments (Figure 1). Hence, we will further investigate the following molecular mechanisms of resistin effects on HCC adhesion to the endothelium by treating the cells with 50 ng/mL of resistin.

### Resistin-induced SK-Hep1 cell adhesions to HUVECs was inhibited by AMPK

**Figure 2:**
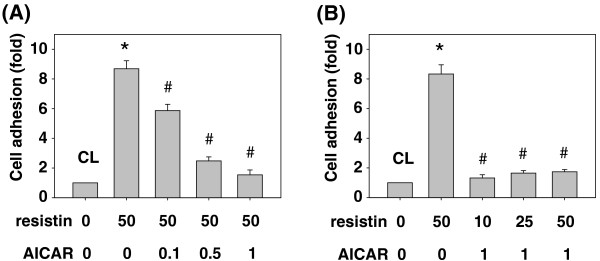
**Resistin-induced SK-Hep1 cell adhesions to HUVECs were inhibited by AMPK. (A)** SK-Hep1 cells were pretreated with AICAR, AMPK activator, at different concentrations (i.e. 0, 0.1, 0.5 and 1 ng/ml) for 1 h. They were then kept as the control (CL) or treated with resistin (50 ng/mL) for 4 h. **(B)** SK-Hep1 cells were pretreated with AICAR at 0 or 1 ng/mL for 1 h. They were then kept as the control (CL) or treated with different concentrations of resistin (i.e. 10, 25 and 50 ng/mL) for 4 h. The bar graphs represent the mean ± SEM from three independent experiments. *, *P* < 0.05 *vs*. control cells. #, *P* < 0.05 *vs.* cells treated with resistin only.

Recently, AMPK, an energy-sensing kinase, was shown to regulate cancer cell metastasis. Hence, we investigated whether resistin-increased adhesions of SK-Hep1 cells to HUVECs are mediated by AMPK. First, SK-Hep1 cells were pretreated with AICAR, AMPK activator, at different concentrations (i.e., 0, 0.1, 0.5 and 1 ng/ml) for 1 h and then kept as the control or treated with resistin (50 ng/mL) for 4 h, and their adhesions to HUVECs were examined. Treatment with only resistin increased the SK-Hep1 cell adhesions to HUVECs, which reached a level approximately 8 times the untreated control. However, pretreatment with AICAR at 0.1, 0.5 and 1 ng/ml resulted in significant decreases on resistin-induced SK-Hep1 cell adhesions to HUVECs in a dose-dependent manner (Figure 2A). Next, SK-Hep1 cells were pretreated with AICAR at 0 or 1 ng/mL for 1 h and then kept as the control or treated with different concentrations of resistin (i.e. 10, 25 and 50 ng/mL) for 4 h. In all three concentration doses of resistin, pretreatment with 1 ng/mL of AICAR inhibited the resistin effects on SK-Hep1 cell adhesions to HUVECs, compared to treatment with resistin-only cells (Figure 2B).

### Resistin induced both ICAM-1 and VCAM-1 expressions in SK-Hep1 cells

**Figure 3:**
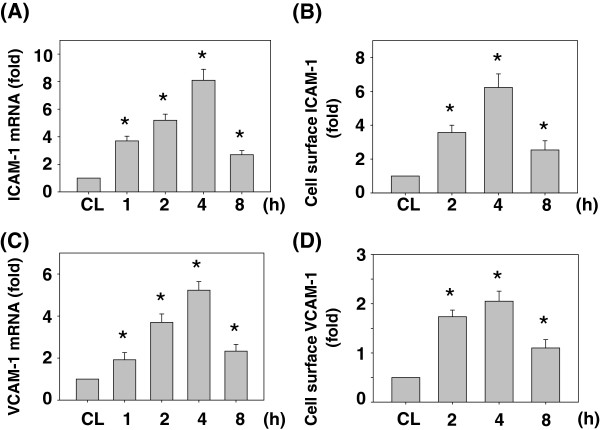
**Resistin induced both ICAM-1 and VCAM-1 expressions in SK-Hep1 cells.** SK-Hep1 Cells were kept as the control (CL) or treated with resistin (50 ng/mL) for 1, 2, 4 and 8 h. They were then analyzed by real-time PCR for ICAM-1 and VCAM-1 mRNA expressions **(A and C)** and ELISA for ICAM-1 and VCAM-1 cell surface protein expressions **(B and D)**. The bar graphs represent the mean ± SEM from three independent experiments. *, *P* < 0.05 *vs*. control cells.

Because cell adhesion molecules have been shown to be critical in cancer cell metastasis, we examined the effect of resistin on the ICAM-1 and VCAM-1 mRNA and cell surface protein expressions in SK-Hep1 cells. SK-Hep1 cells were kept as the control or treated with resistin (50 ng/mL) for 1, 2, 4 and 8 h and then analyzed by real-time PCR for ICAM-1 and VCAM-1 mRNA expressions and ELISA for ICAM-1 and VCAM-1 cell surface protein expressions. Treatment with resistin for 1, 2, 4 and 8 h induced rapid increases (within 1 h) in the ICAM-1 and VCAM-1 mRNA expressions (Figure 3A and C) and cell surface protein expressions (Figure 3B and D), which reached a maximal level compared to the untreated control within 4 h, and then declined but remained elevated after 8 h of treatment.

### The inhibitions of ICAM-1 and VCAM-1 activity and mRNA expression in SK-Hep1 cells attenuated their adhesions to HUVECs

**Figure 4:**
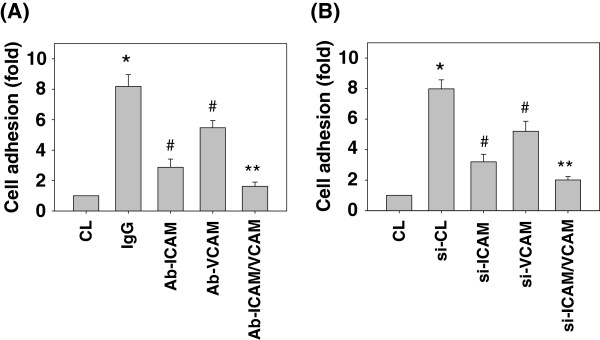
**The inhibitions of ICAM-1 and VCAM-1 activity and mRNA expression in SK-Hep1 cells attenuated their adhesions to HUVECs.** SK-Hep1 cells were pretreated with IgG, ICAM-1, VCAM-1, or both neutralizing antibodies **(A)** or with ICAM-1, VCAM-1, or both specific siRNA **(B)**. They were then kept as the control or treated with resistin (50 ng/mL) for 4 h. The bar graphs represent the mean ± SEM from three independent experiments. *, *P* < 0.05 *vs*. control cells (CL). #, *P* < 0.05 *vs.* cells pretreated with IgG or siCL and then treated with resistin only. **, *P* < 0.05 *vs.* cells pretreated with ICAM-1 or VCAM-1 single neutralizing antibody or siRNA and then treated with resistin.

To investigate the roles of ICMA-1 and VCAM-1 in the adhesions of SK-Hep1 cells to HUVECs, we blocked the ICAM-1 and VCAM-1 activities by using neutralizing antibodies and inhibited their mRNA expressions by using ICAM-1- and VCAM-1-specific siRNAs. SK-Hep1 cells were pretreated with IgG, ICAM-1, VCAM-1, or both neutralizing antibodies. They were then kept as the control or treated with resistin (50 ng/mL) for 4 h. The resistin-increased cell adhesions to HUVECs were attenuated by ICAM-1 or VCAM-1 single-antibody treatment as compared to IgG pretreated cells. Treating the cells with both neutralizing antibodies resulted in enhanced attenuation effects as compared to single-neutralizing antibody pretreated cells (Figure 4A). The inhibitions of resistin-increased SK-Hep1 cell adhesions to HUVECs by blocking ICAM-1 and VCAM-1 activities were substantiated by transfecting the cells with ICAM-1-, VCAM-1-, or both specific siRNAs (40nM), which showed the similar inhibitory effects to the neutralizing antibodies stimulations on resistin-increased SK-Hep1 cell adhesions to HUVECs (Figure 4B). ICAM-1- and VCAM-1-specific siRNAs had 70 ~ 80% blocking effects on their respective protein expressions (data not shown).

### Resistin-induced ICAM-1 and VCAM-1 expressions were mediated by AMPK

**Figure 5:**
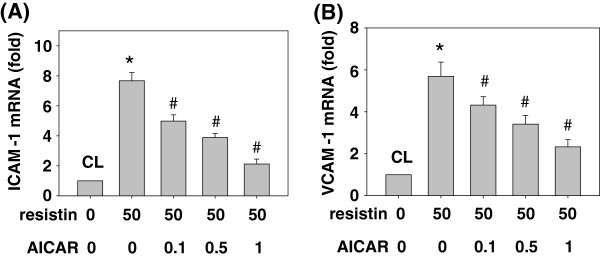
**Resistin-induced ICAM-1 and VCAM-1 expressions were mediated by AMPK.** SK-Hep1 cells were pretreated with AICAR at different concentrations (i.e. 0, 0.1, 0.5 and 1 ng/ml) for 1 h. They were then kept as the control or treated with resistin (50 ng/mL) for 4 h, and their ICAM-1 **(A)** and VCAM-1 **(B)** mRNA expressions were determined by using real-time PCR. The bar graphs represent the mean ± SEM from three independent experiments. *, *P* < 0.05 *vs*. control cells. #, *P* < 0.05 *vs.* cells treated with resistin only.

Next, we determined whether resistin-induced ICAM-1 and VCAM-1 mRNA expressions are mediated through AMPK in SK-Hep1 cells. SK-Hep1 cells were pretreated with AICAR at different concentrations (i.e. 0, 0.1, 0.5 and 1 ng/ml) for 1 h and then kept as the control or treated with resistin (50 ng/mL) for 4 h. Their ICAM-1 and VCAM-1 mRNA expressions were determined by real-time PCR. Treatment with only resistin induced ICAM-1 and VCAM-1 mRNA expressions, which reached a level approximately 8 and 6 times that of the untreated control, respectively. However, pretreatment with AICAR at 0.1, 0.5 and 1 ng/ml resulted in significant inhibitions of ICAM-1 and VCAM-1 mRNA expressions in SK-Hep1 cells (Figure 5).

### AMPK attenuates resistin-increased SK-Hep1 cell adhesions to HUVECs through activating the NF-κB

**Figure 6:**
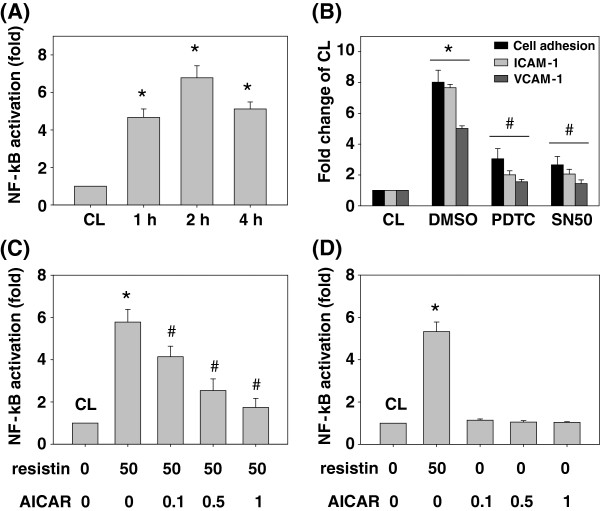
**AMPK attenuated resistin-increased SK-Hep1 cell adhesions to HUVECs through activating the NF-κB. (A)** SK-Hep1 cells were kept as the control or treated with resistin (50 ng/mL) for 1, 2 and 4 h. **(B)** SK-Hep1 cells were kept as the control pretreated with DMSO or NF-κB inhibitors, PDTC or SN50, to inhibit activity. They were then treated with resistin (50 ng/mL) for 4 h. **(C)** SK-Hep1 cells were kept as the control or pretreated with AICAR at different concentrations (i.e. 0, 0.1, 0.5 and 1 ng/ml) for 1 h and then kept as the control or treated with resistin (50 ng/mL) for 4 h. **(D)** SK-Hep1 cells were treated with resistin (50 ng/mL) or AICAR at different concentrations (i.e. 0, 0.1, 0.5 and 1 ng/ml) for 4 h. *, *P* < 0.05 *vs*. control cells. #, *P* < 0.05 *vs.* cells treated with resistin only.

NF-κB is the most important transcription factor in modulating cell adhesion molecule expressions, and also plays a critical role in the resistin-signaling pathway. We investigated whether the inhibitory effects of AMPK on resistin-increased SK-Hep1 cell adhesions to HUVECs were mediated by NF-κB. SK-Hep1 cells were kept as the control or treated with resistin (50 ng/mL) for 1, 2 and 4 h. The NF-κB activations were analyzed by ELISA. Treating the cells with resistin induced rapid activations (within 1 h) of NF-κB, which reached a maximal level in the untreated control within 2 h; it then declined but remained elevated after 4 h of treatment (Figure 6A). SK-Hep1 cells were kept as the control or pretreated with DMSO or NF-κB inhibitors, PDTC (20 μM) or SN50 (50 μg/mL), and then treated with resistin (50 ng/mL) for 4 h. Treating the cells with DMSO/resistin increased the SK-Hep1 cell adhesion to HUVECs and ICAM-1 and VCAM-1 expressions in the same way as our previous data. However, both NF-κB inhibitor treatments inhibited these effects (Figure 6B). Next, SK-Hep1 cells were kept as the control or pretreated with AICAR at different concentrations (i.e. 0, 0.1, 0.5 and 1 ng/ml) for 1 h and then treated with resistin (50 ng/mL) for 4 h, and NF-κB activity was determined by ELISA. Treatment with only resistin induced NF-κB activations, which reached a level approximately 6 times that of the untreated control. However, pretreatment with AICAR at 0.1, 0.5 and 1 ng/ml resulted in significant inhibitions of NF-κB activations in SK-Hep1 cells (Figure 6C). Treatment with AICAR alone at 0.1, 0.5 and 1 ng/ml has no effect on NF-κB activations (compared to untreated control) in SK-Hep1 cells (Figure 6D).

## Discussion

The aim of the present study was to investigate the role of resistin in HCC adhesion to the endothelium and the mechanism underlying the resistin effect. In a series of systematic studies, we have characterized the mechanisms by which resistin regulates the SK-Hep1 cell adhesion to the endothelium through ICAM-1 and VCAM-1 expressions in SK-Hep1 cells; we have found that AMPK activation serves as an antagonist to attenuate this effect. These conclusions were based on the following results: (*i*) Resistin increases the SK-Hep1 cell adhesion to HUVECs. However, AMPK activation in SK-Hep1 significantly attenuates this resistin effect. (*ii*) Resistin-increased SK-Hep1 cell adhesions to HUVECs occur through the induction of ICAM-1 and VCAM-1 mRNA and cell surface protein expressions in SK-Hep1 cells. (*iii*) AMPK activation-attenuated resistin effects on SK-Hep1 cell adhesion to HUVECs are through inhibiting NF-κB activity and following ICAM- and VCAM-1 expressions. Thus, our findings provide insights into the molecular mechanisms by which resistin regulates HCC adhesion to the endothelium in SK-Hep1 cells.

Increasing epidemiological evidence has demonstrated that obesity is associated with a range of cancer types, including HCC [5-8]. In addition to regulate the metabolic homeostasis within the adipose tissue, adipocyte-secreted adipokines have also been indicated to play important role both in cancer risk and cancer mortality [11]. Our study has shown that resistin induces SK-Hep1 cell adhesion to the endothelial cells. Recently, resistin has been implicated to serve as a marker of diagnosis and prognosis in different types of cancer. Lee et al. and Dalamaga et al. indicated that resistin expressions in breast cancer tissues are associated with patient clinicopathological variables [10,24]. Danese et al. clarified that the serum resistin levels are significantly higher in colorectal cancer patients than in healthy controls [1]. However, the precise roles of resistin on cancer development are still unclear. In the present study, we have demonstrated that (a) resistin has no inhibitory effects on the viability of SK-Hep1 cells (see Additional file 1) and (b) resistin has capability to induce the adhesion of SK-Hep1 cell to the endothelial cells and hence we have speculated that resistin might be an important mediator for initiating the HCC metastasis.

The adhesion of cancer cell to the vascular endothelium is a critical step to result in the cancer cell metastasis [25,26]. The expressions of adhesion molecules, including ICAM-1 and VCAM-1, in endothelial cells have been shown to play important roles to mediate this metastatic process [26]. However, our study has shown that resistin induces both ICAM-1 and VCAM-1 expressions in SK-Hep1 cells. Recent studies have indicated that the serum ICAM-1 and/or VCAM-1 are increased in HCC, breast and colorectal cancer [27-31]. However, it has been shown that these elevated adhesion molecules expressions in patients are associated with advanced diseases and might contribute prognostic significance in patients. Our study further demonstrated that the role of both ICAM-1 and VCAM-1 in the SK-Hep1 cells is to promote the SK-Hep1 cell adhesion to the endothelial cells under resistin stimulation. The receptors of ICAM-1 and VCAM-1 are the LFA-1 and VLA4 integrins, respectively, and they are only express in the immune cells, including the leukocytes [32]. Moreover, the elevated soluble serum ICMA-1 and VCAM-1 in patients have also been shown to be associated with the inflammatory reactions within tissues [27-31]. Recently novel metastatic theory has indicated that the cancer cell-leukocyte fusion play potential role in the cancer metastasis [33]. Therefore, the issue about whether resistin-induced HCC adhesion to the endothelium through ICAM-and VCAM-1 in HCC is regulated by leukocytes warrants further exploration.

AMPK is known to be an energy sensor that controls the cellular metabolisms in normal cells [34-38]. However, it has also been indicated that AMPK is a novel therapeutic target for cancer and metabolic diseases [39-41]. Our study has shown that AMPK activation attenuates the resistin-increased SK-Hep1 cell adhesion to the endothelial cells. This data is supporting by recent discoveries that AMPK activation play a critical role in inhibiting cancer metastasis: (a) Taliaferro-Smith et al. demonstrated that AMPK activation by adiponectin inhibits the migration of breast cancer cells [42]; (b) Kin et al. indicated that berberine increases AMPK activity and hence decreases the migration of melanoma cells [20]; (c) Park et al. also demonstrated that berberine inhibits colon cancer migration by activating AMPK [21]. The inhibitory effects of AMPK activation on resistin-induced SK-Hep1 cell adhesion in our study are through adding the AMPK agonist, AICAR. Recent study has shown that resistin inhibit the AMPK phosphorylation to modulate glucose metabolism in human hepatoma cells. Thus, in our study, whether resistin-induced the SK-Hep1 cell adhesion to the endothelial cells is through inhibiting AMPK phosphorylation and activation remains to be determined. In brief, the identification of AMPK activators may be a promising strategy for the development of novel therapeutic drugs for cancer metastasis.

## Conclusion

Our present study has demonstrated that resistin increases SK-Hep1 cell adhesion to the endothelial cells, with concomitant increases in NFκB activity and ICAM-1 and VCAM-1 mRNA, as well as cell surface protein expressions in SK-Hep1 cells. However, these resistin-increased responses are attenuated by AMPK activation. Our findings provide a notion that resistin play an important role in modulating HCC adhesions and suggest AMPK may be a promising target for a therapeutic intervention against HCC metastasis.

## Abbreviations

HCC: Hepatocellular carcinoma; AMPK: AMP-activated protein kinase; ICAM-1: Intercellular adhesion molecule-1; VCAM-1: Vascular cell adhesion molecule-1; AICAR: 5-Aminoimidazole-4-carboxamide 1-β-D-ribofuranoside; HUVEC: Human umbilical vein endothelial cells.

## Competing interests

The authors declare that they have no competing interests.

## Authors’ contributions

C–CY, S-FC and W–HK designed research; C–CY, J-KC, Y-LL, W-EC, and W–HH performed research; C–CY, S-FC and W–HK analyzed data; and S-FC and W–HK wrote the paper. All authors read and approved the final manuscript.

## Pre-publication history

The pre-publication history for this paper can be accessed here:

http://www.biomedcentral.com/1471-2407/14/112/prepub

## Supplementary Material

Additional file 1 **Effect of resistin on cell viability of SK-Hep1.** (A) Cells were kept as controls (CL) or stimulated with resistin at the indicated time periods. Cell viability was assayed by the MTT test. Bar graphs represent folds of CL cells, mean ± standard error of the mean (SEM). *P < 0.05 versus CL. Click here for file
