# Supplementary material for: Activation of AMP-activated protein kinase attenuates hepatocellular carcinoma cell adhesion stimulated by adipokine resistin
Source: BMC Cancer. 2014 Feb 20;14:112. doi: 10.1186/1471-2407-14-112 (PMC3936704; doi:10.1186/1471-2407-14-112)
Supplement: Additional file 1 — Effect of resistin on cell viability of SK-Hep1. (A) Cells were kept as controls (CL) or stimulated with resistin at the indicated time periods. Cell viability was assayed by the MTT test. Bar graphs represent folds of CL cells, mean ± standard error of the mean (SEM). *P < 0.05 versus CL. [file 1471-2407-14-112-S1.pdf]

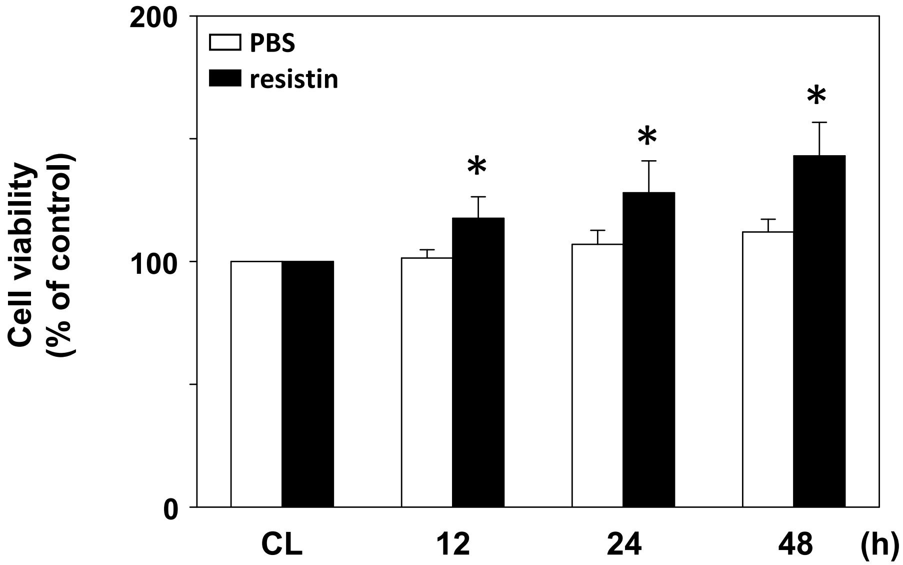

Effect of resistin on cell viability of SK-Hep1. (A) Cells were kept as controls (CL) or stimulated with resistin at the indicated time periods. Cell viability was assayed by the MTT test. Bar graphs represent folds of CL cells, mean ± standard error of the mean (SEM). *$P < 0.05$ versus CL.
